# Explaing users’ technology acceptance through national cultural values in the hospital context

**DOI:** 10.1186/s12913-022-07488-3

**Published:** 2022-01-17

**Authors:** C. Metallo, R. Agrifoglio, L. Lepore, L. Landriani

**Affiliations:** 1 Department of Science and Technology, University of Naples Parthenope, Centro Direzionale –Isola C4, 80143 Naples, Italy; 2 Department of Business and Economics, University of Naples Parthenope, Naples, Italy; 3 Department of Law, University of Naples Parthenope, Naples, Italy

**Keywords:** Health information technology, Technology Acceptance Model, National culture

## Abstract

**Background:**

Current research demonstrates that health information technology can improve the efficiency and quality of health services. However, many implementation projects have failed due to behavioural problems associated with technology usages, such as underuse, resistance, sabotage, and even rejection by potential users. Therefore, user acceptance was one of the main factors contributing to the success of health information technology implementation. However, research suggests that behavioural models do not universally hold across cultures.

The present article considers national cultural values (power distance, uncertainty avoidance, individualism/collectivism, masculinity/femininity, and time orientation) as individual difference variables that affect user behaviour and incorporates them into the Technology Acceptance Model (TAM) as moderators of technology acceptance relationships. Therefore, this research analyses which national cultural values affect technology acceptance behaviour in hospitals.

**Methods:**

The authors develop and test seven hypotheses regarding this relationship using the partial least squares (PLS) technique, a structural equation modelling method. The authors collected data from 160 questionnaires completed by clinicians and non-clinicians working in one hospital.

**Results:**

The findings show that uncertainty avoidance, masculinity/femininity, and time orientation are the national cultural values that affect technology acceptance in hospitals. In particular, individuals with masculine cultural values, higher uncertainty avoidance, and a long-term orientation influence behavioural intention to use technology.

**Conclusion:**

The bureaucratic model still decisively characterises the Italian health sector and consequently affects the choices of firms and workers, including the choice of technology adoption. Cultural values of masculinity, risk aversion, and long-term orientation affect intention to use through social norms rather than through perceived utility.

**Supplementary Information:**

The online version contains supplementary material available at 10.1186/s12913-022-07488-3.

## Background

Current research demonstrates that health information technology (HIT) can improve patient safety and healthcare quality, yielding cost savings and reducing medical error rates [1]. However, many implementation projects within the health sector have failed or been abandoned [2]. One of the major factors leading to failure is the inadequate understanding of how clinicians and users react to implemented HIT [3]. For example, Bhattacherjee and Hikmet [4] investigated behavioural problems associated with HIT usage, such as physician resistance, showing that there has been little research on why or how behavioural problems occur. Therefore, several barriers must be overcome for HIT implementation in a hospital [5–7].

Technologies usage in medical settings frequently collides with users’ resistance, even if they can benefit from their use [4]. HIT implementation can be linked to underuse, resistance, and sabotage by users, which means many difficulties to achieve the innovation potential imbued in the technology [8]. Therefore, the research considers user acceptance as an important factor contributing to HIT success. In line with this, the Technology Acceptance Model (TAM) [9, 10] is the framework most frequently used to explain users’ acceptance behaviour towards technology. According to the TAM, the behavioural intention to use technology is determined by two beliefs ([10], p. 320): the perceived usefulness (PU), “the degree to which a person believes that using a particular system would enhance his or her job performance”; and the perceived ease of use (PEOU), “the degree to which a person believes that using a particular system would be free of effort”. Several studies have aimed to improve the TAM model, such as the inclusion of subjective norms (SNs), “the person’s perception that most people who are important to him think he should or should not perform the behaviour in question” ([11], p. 302).

Melas et al. [12] highlighted that the TAM is a good predictor of HIT acceptance. In particular, the reviews by Yarbrough and Smith [13] and Holden and Karsh [3] on the utilisation of the TAM in the healthcare context shown that studies are heterogeneous and attach little importance to moderators, despite TAM research’s findings [12]. In addition, research suggests that behavioural models tend to differ across cultures [15–17]. Thus, Lin [14] explored national cultural differences as moderators of nurses’ perspectives on HIT acceptance. Therefore, a research stream emphasises national cultural values as individual difference variables that can play a moderating role within TAM relationships [18, 19, 14].

The current study is part of this research stream and analyses how national culture impacts technology acceptance in healthcare.

Hofstede defines culture as “the collective programming of the mind which distinguishes the members of one human group from another” [15]. To date, the most popular conceptualisation of national culture has been Hofstede’s [15, 20] taxonomy and subsequent extensions [21], describing culture along the following dimensions: power distance, uncertainty avoidance, individualism-collectivism, masculinity-femininity, and time orientation. According to Hofstede [20] and Hofstede and Bond [21], the cultural dimensions are the following:

1. power distance refers to how people accept the relationship of hierarchical power, that is, the degree of inequality that exists within society;
2. uncertainty avoidance is the degree to which a society is averse to uncertainty and ambiguity;
3. individualism-collectivism represents a preference for a society that emphasises individual goals (individualism), while collectivism is a society where people are integrated into groups;
4. masculinity-femininity refers to the preference for ambition or material success, that is, a society that stresses different gender roles (high masculinity) vs low preference (femininity);
5. time orientation is the degree to which people emphasise future benefit (long-term orientation) or stress immediate rewards (short-term orientation).

Hence, this study considers national cultural values (power distance, uncertainty avoidance, individualism/collectivism, masculinity/femininity, and time orientation) as individual difference variables that affect user behaviour and incorporates them into the TAM as moderators of technology acceptance relationships. Therefore, the research question of this article is as follows: which national cultural values promote users’ acceptance of technology in hospitals?

### Research model

Research has shown that masculine or feminine values affect relationships in the TAM [18, 22, 23, 14]. Thus, some scholars [18, 23] have stressed that differences in the perception and use of technology might be affected by psychological gender, the prevalence of masculinity or femininity values within society [24, 25]. A working environment with high masculinity is generally characterised by values such as competitiveness and material success, and people tend to be goal-oriented; research has shown that such values can be linked to the individual perceived that technology might improve job performance (perceived usefulness) [26, 27, 18, 14]. Therefore, individuals who espouse masculine cultural values tend to give more emphasis on perceived usefulness than individuals who espouse feminine cultural values.

Moreover, Srite and Karahanna [18] observed that individuals who espouse feminine cultural values are most interested in the quality of work-life and the creation of pleasant and less frustrating work values [25]. These people are inclined to adopt technology that requires little or no effort and, thus, they tend to emphasise perceived ease of use, that is, on the extent to which using the technology is effort-free and easy to use [28]. Hence, this study proposes the following hypothesis:

Hypothesis 1: The espoused national cultural value of masculinity/femininity moderates the relationship between perceived usefulness and behavioural intention as well as the relationship between perceived ease of use and behavioural intention.

Masculinity-femininity can also influence the relationship between social norms and behavioural intention to use [26]. According to Bollinger and Hofstede [29], a masculine culture is characterised by competition and success; thus, societies with higher masculinity levels stand out by a desire for material goods and the importance of social status. Bollinger and Hofstede [29] consider Italy a society with a masculine culture since Italian people attach importance to a search for competitive results, affecting technology use behaviour [18, 14]. In highly bureaucratised environments, such as hospitals, the prevailing social behaviour conforms to the majority without undertaking innovative paths [30]. Wooten and Crane [31] observed that clan control mechanisms are developed (congruence of objectives and shared values) to coordinate the individual’s behaviour; consequently, in hospitals, the clan mechanism facilitates the generation of consensus decision making. The bureaucratised environment and the goal orientation of physicians’ work can make the hospital management or peer pressure influence whether a physician will adopt a technology [19]. Thus, pressure in the pursuit of success leads people to consider the social influence of supervisors or peers. Some studies have investigated the cultural value of individualism/collectivism within the relationship between subjective norms and behavioural intention to use [32, 18, 33–35]. In collectivist cultures, people are significantly influenced by the group and take into account of opinions of others, mainly to satisfy the need for approval from the group [24]. In contrast, individuals who espouse individualistic cultural values tend to be self-oriented, believe in individual decisions, and are more independent and less loyal to the group than people from collectivistic cultures [27]. Therefore, in a high-individualism cultural environment, people are less concerned with the opinions of others [18]. Thus, the influence of subjective norms on behavioural intention is stronger in a collectivistic culture [32]. This leads to the following hypothesis:

Hypothesis 2: The relationship between subjective norms and behavioural intention to use is moderated by the espoused national cultural values of masculinity/femininity and of individualism/collectivism such that the relationship is stronger for individuals with espoused masculine and collectivistic cultural values.

Scholars have suggested that the cultural value of power distance might moderate the relationship between subjective norms and behavioural intention to use [36, 18, 37–39]. People with higher power distance levels tend to be more careful in conforming to the opinions of their superiors [40, 19]. Therefore, these individuals would be more influenced by social norms when deciding whether to adopt technologies [18, 39].

Furthermore, individuals with high uncertainty avoidance values tend to respect rules and adapt to others’ opinions, supervisors, and peers, to reduce this uncertainty [18, 40, 19]. In this context, subjective norms may reduce uncertainty when peers, supervisors, or friends share personal experiences and perceptions of the technology [18]. Thus, social norms might serve as determinants of behaviour for individuals with higher uncertainty avoidance values than those with uncertainty avoidance values. Thus, the research hypothesizes as follows:

Hypothesis 3: The relationship between subjective norms and behavioural intention to use is moderated by the espoused national cultural values of power distance and of uncertainty avoidance such that the relationship is stronger for individuals with higher espoused power distance and uncertainty avoidance cultural values.

Time orientation (short- vs long-term orientation) may affect users’ perception of technology and, particularly, their behavioural intention to use technology [41–43, 14, 28]. Cultures with a long-term orientation tend to foster trust and behaviours such as thrift or perseverance towards future rewards; in contrast, cultures with a short-term orientation tend to exhibit a focus on achieving quick results [43].

People with long-term orientation tend to be bonded to current working practices and the routine and values intrinsic in their specific tasks [15]. Indeed, Hofstede [15] highlighted that adaptiveness is the principal work value of long-term orientation. A level low on this dimension, for example, indicates the preference to preserve traditions and norms consolidated over time. Instead, a level high on long-term orientation indicates a more practical approach and, thus, the capacity to adapt traditions with ease to changing conditions. Some scholars have shown that cultures with high long-term orientation levels are more likely to adopt new technologies [44]. Moreover, long-term orientation also increases the imitation effect [45]: traditions can be an obstacle to change, but, one time a change is socially accepted, it is rapidly implemented. Thus, the long-term orientation would more affect intention to use technology than short-term orientation for individuals.

Hypothesis 4: Individuals who are long-term oriented will exert more of an influence on behavioural intention to use technology.

**Fig. 1:**
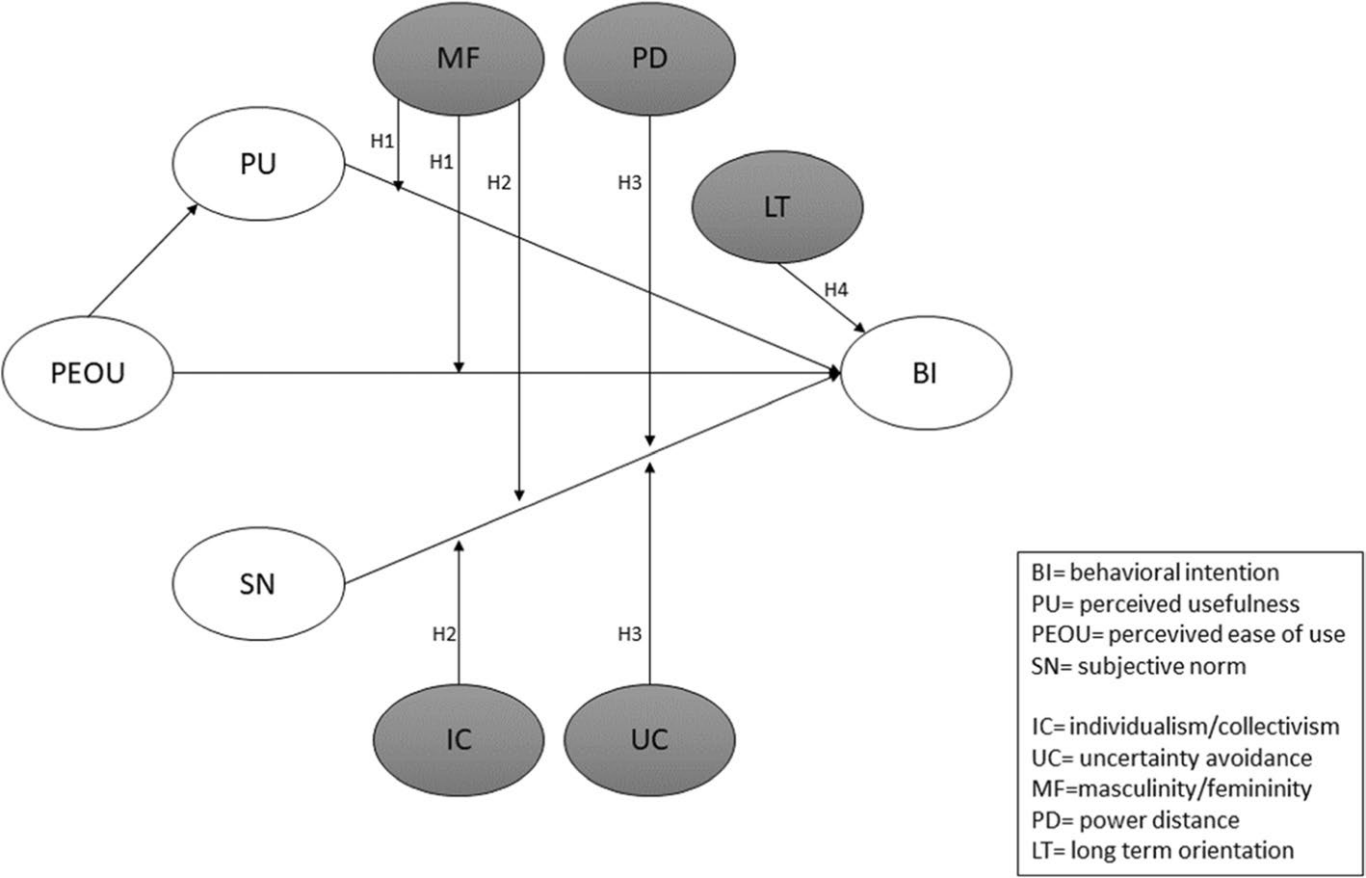
The proposed research model

The research model is shown in Fig. 1.

## Methodology

The proposed model was tested through a quantitative methodology. A survey method for data collection was used to gather data from September to December 2019. Data were collected by administering a structured questionnaire in the Italian language to 400 healthcare workers (clinicians and non-clinicians) in one of the largest hospitals in southern Italy. A pilot test was conducted with three healthcare managers to assess the consistency of translated items.

Of the 300 administered questionnaires, 190 were returned completed (response rate of 63%). All the collected data were checked for consistency to minimise data entry errors. As a result, 160 valid responses were included.

The questionnaire was divided into two sections. The first section was intended to capture the profile of the survey respondents (age, gender, education level, and IT experience), while the second section contained 32 survey questions derived from the existing IS literature. In particular, BI and SN were measured using Venkatesh and Davis’s [46] two-item scale, while PU and PEOU were measured using Venkatesh and Davis’s [46] four-item scale. Finally, cultural orientation variables, such as MF, IC, PD, UC, and LT, were measured using Baptista and Oliveira’s [47] twenty-item scale. All variables were measured on a seven-point scale, ranging from ‘strongly disagree’ [1] to ‘strongly agree’ [7].

## Data analysis and results

The data analysis was performed using the partial least squares (PLS) method, a structural equation modelling technique used in IS research [48]. Consistent with prior IS research [49], data was perfomed through a PLS-SEM application (XLSTAT) by using a reflective measurement model (i.e., indicators of a construct are considered to be caused by that construct). Using XLSTAT, we first established the psychometric validity of the scales used through the construct reliability -Cronbach’s alpha (α) and composite reliability (ρc)- and discriminant validity -Average Variance Extracted (AVE)-.

Regarding the construct reliability, we noted that both α and ρc must be greater than 0.80 (BI: α = 0.89, ρc = 0.93; PEOU: α = 0.92, ρc = 0.94; PU: α = 0.91, ρc = 0.94; SN: α = 0.90, ρc = 0.92; MF: α = 0.97, ρc = 0.98; IC: α = 0.87, ρc = 0.91; PD: α = 0.88, ρc = 0.92; UC: α = 0.89, ρc = 0.92; and LT: α = 0.92, ρc = 0.94) to indicate good reliability.

Regarding the discriminant validity, we noted that the square root of the AVE for each construct (diagonal of Table 1) is larger than the correlations with other constructs. Table 1 shows the comparison between the AVE and construct correlations.

**Table 1:** Discriminant validity (*N* = 190)

	**AVE**	**PEOU**	**PU**	**SN**	**IC**	**UC**	**LT**	**MF**	**PD**	**BI**
PEOU	0.803	**0.896**								
PU	0.788	0.830	**0.888**							
SN	0.762	0.873	0.830	**0.873**						
IC	0.723	0.724	0.770	0.758	**0.850**					
UC	0.748	0.746	0.697	0.715	0.813	**0.865**				
LT	0.799	0.757	0.755	0.768	0.765	0.787	**0.894**			
MF	0.898	0.073	0.000	0.008	0.077	0.139	0.067	**0.948**		
PD	0.742	0.548	0.500	0.560	0.566	0.560	0.609	0.176	**0.861**	
BI	0.817	0.769	0.731	0.848	0.712	0.678	0.744	0.033	0.530	**0.904**

Moreover, we also noted that all items have factor loadings of 0.70 or greater on their corresponding constructs, as well as they load to a low extent on the other ones, so confirming the discriminant validity. The Additional file 1 displays the scales used in the study and the related factor loadings.

After verifying the constructs’ reliability and the discriminant validity, data analysis was performed using the PLS technique. Table 2 shows the findings of the PLS analysis.

**Table 2:** PLS estimations (*N* = 190)

	**(I)** **PU**	**(II)** **BI**	**(III)** **BI**
*Independent variables:*			
PEOU	0.825***	0.087	0.211
PU		0.063	0.089
SN		0.715***	0.560**
LT			0.181**
*Moderating variables:*			
MFxPU			-0.501
MFxPEOU			-0.717
MFxSN			1.212*
ICxSN			-2.203**
PDxSN			-0.568
UCxSN			2.358**
**R**^**2**^	0.681	0.734	0.759
**Adjusted R**^**2**^	0.681	0.729	0.737

^***^ p ≤ 0.001, ** p ≤ 0.005, and * p ≤ *0.010*

Table 2 shows the findings of the PLS analysis. The three models reported in Table 2 (I, II, and III) show the effects of the independent and moderator variables on dependent variables (PU and BI). In particular, Table 2 shows the effects of the PEOU independent variable on the PU dependent variable (Model I), the effects of the TAM variables and SN on the BI dependent variable (Model II), and the effects of the independent (PEOU, PU, SN, and LT) and moderating variables (MF, IC, PD, and UC) on the BI dependent variable (Model III).

As shown in Table 2, the proposed research model explains approximately 76% of the variance, which SN (β = 0.560, *p* ≤ 0.005) and LT (β = 0.181, *p* ≤ 0.005) significantly determine BI independent variables. Thus, hypothesis H4 is supported by the data.

Unlike, findings show that PU and PEOU do not affect BI, thus H1 isn’t supported by the data. Furthermore, the findings of the PLS analysis also show that MF (β = 1.212 m *p* ≤ 0.010), IC (β = -2.203 m *p* ≤ 0.005), and UC (β = 2.358, *p* ≤ 0.005) moderate the relationship between SN and BI, while PD doesn’t moderate this relationship. However, unlike we hypothesised, the explanatory contribution of IC is negative; thus, H2 and H3 are only partially supported by the data.

## Discussion

The study analysed how national cultural values (power distance, uncertainty avoidance, individualism/collectivism, masculinity/femininity, and time orientation) affect technology acceptance in hospitals.

In order to test how national cultural values affect the technology acceptance model, we first tested the explanatory contributions of the leading technology model variables revealed by the IS literature, such as PU, PEOU, and SN, to BI. Our findings have shown that PU and PEOU do not affect BI. Empirical investigations on technology acceptance model testing have shown that PEOU does not always influence BI, while the explanatory contribution of PU to BI is often found. In our study, PU does not affect BI simply because using new information technology is mandatory and therefore not linked to its characteristics and usefulness. In other words, in Italian public health, the active involvement of those who will have to use new technologies in the innovation process is not envisaged as they are required to comply with the law and not choose to improve their performance [50]. This pattern is typical of bureaucracy [7]. In particular, in the context of Italian public hospitals, the strong and rooted bureaucratic culture amplifies the effect of national cultural values on the methods of using new technologies. In this regard, several studies [51–53] highlighted the relationships between national culture and bureaucratic culture, highlighting precisely how there is a possible overlap and emphasis between them. In other words, bureaucracy can be understood as a real manifestation of the cultural model prevalent in a specific nation, a sort of output which, at the same time, also acts as a driver or input to strengthen and consolidate the same clutural values.

In general, bureaucracy is characterised by adherence to written laws, depersonalisation of working relationships, command line hierarchy, and the separation of actions and consequences [54]. In reality, bureaucracy manifests itself in Italy than in other countries by ignoring the connection between individual action and the outcome, both individually and collectively [55]. Furthermore, the bureaucratic rationale is compounded since the healthcare sector is a professional bureaucracy linked to the special abilities held by some categories, such as physicians, nurses, and others. Organisations in this sector, such as hospitals, operate as a clan, a closed structure, which impedes creativity and change. As a consequence, the bureaucratic context is strengthened, forming a vicious cycle [31].

Furthermore, our findings have also shown that SN positively affects BI. This result is consistent with the existing TAM literature [18]. As noted above, the bureaucratic context of Italian healthcare is characterised by processes of isomorphism, which discourage innovation or the adoption of new tools in the single hospital, especially because innovation processes are guided by a top-down orientation, where hospitals also suffer from an almost centralised purchasing process, which severely limits their autonomy. The working climate, in this scenario, influences the BI and the behaviour of use in the conformist direction [56].

Furthermore, since the 1990s, when the healthcare system was reforming according to new public management, the emphasis has been solely on efficiency and productivity, resulting in the standardisation of individual operator activity [57]. Furthermore, the later-introduced compensation and incentive systems have mirrored this strategy, flattening individual performance [58].

Regarding the cultural variables, our findings have shown that masculinity/femininity and uncertainty avoidance moderate the relationships between subjective norms and behavioural intention to use.

Masculine/feminine values had a significant moderating effect on the relationship between subjective norms and behavioural intention to use such that this relationship was stronger for masculine cultures. In particular, our findings show that the prevalence of a masculine culture is linked to an incessant search for results from a competitive perspective, and this decisively affects the way technology is used [18, 14]. As already highlighted, in highly bureaucratised environments, the prevailing social behaviour advises not taking innovative paths but conforming to the majority, thus having an external or contextual influence on the behaviour of use [30]. In addition, the clan logic, which is characteristic of masculinity, expresses itself as obedience to the will of those who administer the institution [31].

A *mechanism copy* logic predominates in the healthcare context and introduces changes and innovation [50]. This is in line with the professional bureaucracy and stems in Italy from the legal approach for reforms [58].

Furthermore, our results have also shown that the individualism/collectivism cultural variable significantly moderates the relationship between SN and BI; however, inconsistent with our hypothesis, the explanatory contribution is negative. Our findings show that the prevalence of individualistic cultural value leads healthcare workers to obtain advantages from aligned solutions, giving up space of autonomy. Therefore, it is not surprising that in the Italian context under investigation, the effect of SN on BI diminishes as a collectivist culture grows. In fact, in the competitive search for results and performance, the collectivist approach is not consistent as it would act as a brake on the choice of different methods of using the technology. In other words, collectivist culture negatively affects BI because it hinders the search for individual performance improvement.

In general, health organisations are confirmed as an individualistic context in which the individual adopts behaviours aimed at optimising personal goals concerning those of the organisation [59]. In particular, this is achieved by adhering to the organisation’s rules and procedures. As confirmed by other studies, the ease of use facilitates the acceptance of new technology; that is, it is the decisive factor in the collectivist approach [14, 42, 60].

Regarding uncertainty avoidance cultural values, risk aversion prevails, which, as seen from the data, increases the effect of social influence on user behaviour. The different actors do not work in dynamic and incentive contexts, so there are no benefits in changing the status quo. In the health sector, in particular, the dominant culture provides risk aversion, i.e., the reduction of uncertainty for achieving a goal [14, 61, 62]. As already highlighted, in Italy, the prevalence of the bureaucratic model induces standardised behaviour [63].

Finally, our findings have shown that long-term orientation positively affects users’ behavioural intention to use technology. The operators are permanent employees of the hospitals and, therefore, will work in the same context for a long time. Furthermore, they know that the choices of adopting a new technology concern the long term and that, as such, they are difficult to change in the short term. For this reason, the sooner they learn to use it and use it, the greater the benefits will be for them; thus, only an orientation towards stability (and, therefore, the long term) can help to favour the spread of new tools. This result is not confirmed in the literature in similar contexts [14, 46] since, in previous studies, the orientation of professionals towards the goal in the short term did not influence the long-term approach.

## Research implications, conclusions and limits

This research demonstrates how Hofstede’s cultural model contributes to highlighting variables capable of increasing the success of the TAM, in its original dimension, concerning the intention to use new technology.

Overall, this will help improve the effectiveness of the implementation processes of new technologies, thus reducing waste of time and resources and failures and rejections.

The analysis was conducted in the Italian hospital healthcare context, which showed some peculiarities [64]. In this context, it is possible to find several studies that have applied the different models individually [65–67], but there have been few attempts to integrate the two [14].

These considerations have at least two implications: one on the theoretical level and the other on the managerial level.

From the theoretical point of view, if the integration of the Hofstede model with the TAM has helped improve its explanatory capabilities, the need for further integration is also highlighted, i.e., with cultural models that consider the sectorial peculiarities in which the analysis occur. As seen from the results, it is precisely the intrinsic characteristics of the Italian healthcare context that determine the possible explanation of the use methods, albeit mediated by Hofstede’s approach. In other words, in the context of national culture, a decisive role is played, also in this case in an integrated way, by the operational particularities of the sector in which the actors involved in the decisions of use operate [68].

In summary, it can be said that the characteristics of the bureaucratic approach, which still decisively characterises the Italian public administration and the health system, are the key factors in guiding the choices of firms and workers [69, 70].

On closer inspection, however, it is a cultural variable and therefore able to interact well with both the Hofstede model and the TAM, albeit at a different and more operational level. The first reason is that it affects that delicate boundary sphere between the values of the individual and the values of the company in which and for which he works; and the second reason is that the intention of use is, in the examined context, more guided by the social norm, understood in a broad sense, concerning perceived utility.

Future research could consider other variables, such as the OCAI model [71] widely used in the healthcare context [72, 62, 7, 51, 73], and investigate the possible role played by bureaucratic culture as a factor that influence the relations between national cultural values and the use of new technologies. Some of these studies highlighted differences between public hospitals and private hospitals, suggesting, among others, the explanatory role of state ownership as a decisive variable in influencing the success of information systems.

Additionally, from a managerial point of view, the usefulness of the Hofstede model in supporting the implementation of processes for the adoption of new technologies is confirmed. In this sense, the data also suggest, in the health sector, the importance for the legislature and management to consider the cultural characteristics of the context in which innovation is going to be placed before selecting new technologies in order to avoid the first failure and then the abandonment of new technologies, as known in the doctrine.

Furthermore, while not discounting the importance of economic convenience or financial limitations in the introduction of new technology [73, 74], the study demonstrates that management must also recognise the involvement of primarily male or female staff and the various types of wards.

Finally, the management entrusted with implementing the new technology, therefore, should clarify in advance that this is an irreversible choice, at least in the short term, if it wants to make the adoption process more efficient.

Furthermore, this variable is consistent with the highly bureaucratic scenario that has always characterised Italian healthcare and, more generally, state institutions [75, 76]. In this context, cultural values of masculinity, risk aversion, and long-term orientation that the findings show are factors capable of affecting intention of use further suggest that managers observe the field of action in advance using a compatible perspective. For example, technology could be modelled more from the user’s perspective.

There are several limitations of the present work. The analysed sample has a low number of observations, so the results are not generalisable; furthermore, considering only the Italian scenario, reference is made to the prevailing cultural model in that country. It may be interesting to extend this survey in future research, especially to culturally distant national contexts.

## Supplementary Information

**Additional file 1.** The Measurement Scales and Factor loadings.

## Data Availability

The datasets used and analysed during the current study are available from the corresponding author on reasonable request.

## Abbreviations

HIT: Health information technology
TAM: Technology Acceptance Model
PU: Perceived usefulness
PEOU: Perceived ease of use
SN: Subjective norm
PD: Power distance
UC: Uncertainty avoidance
IC: Individualism-collectivism
MF: Masculinity-femininity
LO: Long-term orientation
