# Supplementary material for: Explaing users’ technology acceptance through national cultural values in the hospital context
Source: BMC Health Serv Res. 2022 Jan 17;22:84. doi: 10.1186/s12913-022-07488-3 (PMC8764785; doi:10.1186/s12913-022-07488-3)
Supplement: Supplementary file 1 — Additional file 1. The Measurement Scales and Factor loadings. [file 12913_2022_7488_MOESM1_ESM.docx]

**Additional file 1. The Measurement Scales and Factor loadings**

| **Scale** | **Item** | **Loading** |
| --- | --- | --- |
| *Behavioral intention (BI)* | BI1. Assuming I have access to the system, I intend to use it. | 0.919 |
|  | BI2. Given that I have access to the system, I predict that I would use it. | 0.911 |
| *Perceived Usefulness (PU)* | PU1. Using the system improves my performance in my job. | 0.886 |
|  | PU2. Using the system in my job increases my productivity. | 0.883 |
|  | PU3. Using the system enhances my effectiveness in my job. | 0.894 |
|  | PU4. I find the system to be useful in my job. | 0.888 |
| *Perceived Ease of Use (PEOU)* | PEOU1. My interaction with the system is clear and understandable. | 0.883 |
|  | PEOU2. Interacting with the system does not require a lot of my mental effort. | 0,906 |
|  | PEOU3. I find the system to be easy to use. | 0.894 |
|  | PEOU4. I find it easy to get the system to do what I want it to do. | 0.902 |
| *Subjective Norm (SN)* | SN1. People who influence my behavior think that I should use the system. | 0.868 |
|  | SN2. People who are important to me think | 0.865 |
| *Individualism-collectivism (IC)* | IC1. Being accepted as a member of a group is more important than having autonomy and independence. | 0.810 |
|  | IC2. Group success is more important than individual success. | 0.876 |
|  | IC3. Being loyal to a group is more important than individual gain. | 0.860 |
|  | IC4. Individual rewards are not as important as group welfare. | 0.854 |
| *Uncertainty avoidance (UC)* | UC1. Rules and regulations are important because they inform workers what the organization expects of them. | 0.825 |
|  | UC2. Order and structure are very important in a work environment. | 0.881 |
|  | UC3. It is better to have a bad situation that you know about, than to have an uncertain situation which might be better. | 0.883 |
|  | UC4. People should avoid making changes because things could get worse. | 0.870 |
| *Long-short term (LT)* | LT1. Respect for tradition is important for me. | 0.896 |
|  | LT2. I work hard for success in the future. | 0.899 |
|  | LT3. Traditional values are important for me. | 0.933 |
|  | LT4. I plan for the long term. | 0.846 |
| *Masculinity- femininity (MF)* | MF1. It is preferable to have a man in a high-level position rather than a woman. | 0.969 |
|  | MF2. Solving organizational problems requires the active forcible approach which is typical of men | 0.962 |
|  | MF3. It is more important for men to have a professional career than it is for women to have one | 0.956 |
|  | MF4. Women do not value recognition and promotion in their work as much as men do. | 0.901 |
| *Power distance (PD)* | PD1. Managers should make most decisions without consulting subordinates. | 0.805 |
|  | PD2. Manager should not ask subordinates for advice, because they might appear less powerful. | 0.861 |
|  | PD3. Decision making power should stay with top management in the organization and not delegate to lower-level employees. | 0.912 |
|  | PD4. Employees should not question their manager’s decision. | 0.864 |
